# Maslinic Acid Attenuates Ischemia/Reperfusion Injury-Induced Myocardial Inflammation and Apoptosis by Regulating HMGB1-TLR4 Axis

**DOI:** 10.3389/fcvm.2021.768947

**Published:** 2021-11-10

**Authors:** Qi Li, Mengping Xu, Zhuqing Li, Tingting Li, Yilin Wang, Qiao Chen, Yanxin Wang, Jiaxin Feng, Xuemei Yin, Chengzhi Lu

**Affiliations:** ^1^School of Medicine, Nankai University, Tianjin, China; ^2^Department of Cardiology, Tianjin First Center Hospital, Tianjin, China; ^3^Department of Cardiology, The First Center Clinic College of Tianjin Medical University, Tianjin, China

**Keywords:** maslinic acid, myocardial ischemia/reperfusion injury (MI/RI), high mobility group box 1 (HMGB1), apoptosis, inflammation

## Abstract

**Aims:** The inflammatory response and apoptosis are the major pathological features of myocardial ischemia/reperfusion injury (MI/RI). Maslinic acid (MA), a natural pentacyclic triterpene with various bioactivities, plays critical roles in the multiple cellular biological processes, but its protective effects on the pathophysiological processes of MI/RI have not been extensively investigated. Our study aimed to determine whether MA treatment alleviate ischemia/reperfusion (I/R)-induced myocardial inflammation and apoptosis both *in vitro* and *in vivo*, and further reveal the underlying mechanisms.

**Methods and results:** An MI/RI rat model was successfully established by ligating the left anterior descending coronary artery and H9c2 cells were exposed to hypoxia/reoxygenation (H/R) to mimic I/R injury. In addition, prior to H/R stimulation or myocardial I/R operation, the H9c2 cells or rats were treated with varying concentrations of MA or vehicle for 24 h and two consecutive days, respectively. In this study, our results showed that MA could obviously increase the cell viability and decrease the cardiac enzymes release after H/R *in vitro*. MA could significantly improve the H/R-induced cardiomyocyte injury and I/R-induced myocardial injury in a dose-dependent manner. Moreover, MA suppressed the expression of inflammatory cytokines (tumor necrosis factor alpha [TNF-α, interleukin-1β [IL-1β and interleukin-6 [IL-6]) and the expressions of apoptosis-related proteins (cleaved caspase-3 and Bax) as well as increased the levels of anti-apoptotic protein Bcl-2 expression both *in vitro* and *in vivo*. Mechanistically, MA significantly inhibited nuclear translocation of nuclear factor-κB (NF-κB) p65 after H/R *via* regulating high mobility group box 1 (HMGB1)/toll-like receptor 4 (TLR4) axis.

**Conclusion:** Taken together, MA treatment may alleviate MI/RI by suppressing both the inflammation and apoptosis in a dose-dependent manner, and the cardioprotective effect of MA may be partly attributable to the inactivation of HMGB1/TLR4/NF-κB pathway, which offers a new therapeutic strategy for MI/RI.

## Introduction

Myocardial infarction (MI) persists as a leading cause of high morbidity and mortality worldwide (1). While timely primary percutaneous coronary intervention (PCI) or the thrombolytic agents are considered to be the optimal treatment for salvaging ischemic myocardium, the process of reperfusion may also cause irreversible damage to the ischemic myocardium, which is generally termed as myocardial ischemia/reperfusion injury (MI/RI) (2). The pathogenesis of MI/RI is an extremely complex process which results in the cardiac contractile dysfunction, reperfusion arrhythmia, myocardial stunning, and lethal reperfusion injury (3, 4). Therefore, fully understanding the mechanism of MI/RI and exploring the safe and effective drugs are still the focuses of intense research.

The accumulating evidence, such as ours, has demonstrated that the inflammatory response and cardiomyocyte apoptosis play significant roles during MI/RI, which were associated with toll-like receptor 4 (TLR4)/nuclear factor-κB (NF-κB) signaling pathway activation (5–7). Therein, inflammation might be an initial factor of MI/RI, which is observed during the whole pathophysiological processes of ischemia and reperfusion. An inflammation aggravates myocardial damage by increasing the production of inflammatory cytokines, such as tumor necrosis factor-α (TNF-α), interleukin-1β (IL-1β), and interleukin-6 (IL-6) during post-ischemia/reperfusion (I/R) (8, 9). Meanwhile, the acute inflammatory response and infiltration of the inflammatory cells in myocardium also prompt cardiomyocyte apoptosis, which is primarily triggered or accelerated during I/R and partially contributes to overall cardiomyocyte death (9, 10). On the other hand, high mobility group box 1 (HMGB1), a non-histone DNA binding protein, has been found to be passively released by the necrotic cardiomyocytes in response to ischemia and identified as a mediator of inflammation and apoptosis in MI/RI (11, 12). TLR4, a member of the TLR family, can be stimulated by HMGB1, and activation of TLR4 then promotes the activity of NF-κB through the myeloid differentiation factor 88 (MyD88)-dependent pathway. The previous studies have demonstrated that HMGB1-TLR4-MyD88-NF-κB signaling pathway plays a substantial role in mediating the inflammatory response and cardiomyocyte apoptosis following MI/RI (13–15). Thus, finding a safe and reliable treatment to inhibit the inflammatory response and cardiomyocyte apoptosis through mediating HMGB1-TLR4 axis may protect the cardiomyocytes from reperfusion injury.

Maslinic acid (MA), a natural pentacyclic triterpene, can be found in a variety of natural sources, such as Olea europaea and a variety of Asian medicinal plants (16, 17). It is reported that MA possesses a variety of biological properties, such as antioxidant, anti-inflammatory, antidiabetic, antitumor, cardioprotective, and neuroprotective effects (17–22). The recent studies demonstrated that MA exerts anti-inflammatory effect by regulating the TLR4-MyD88 and NF-κB pathways in lung injury (18, 19). In addition, MA reduced the release of HMGB1, which is being proved in the lipopolysaccharide (LPS)-activated human umbilical vein endothelial cells and a cecal ligation and puncture-induced sepsis mouse model (20). In cardiovascular disease, MA also inhibited isoprenaline-induced cardiotoxicity in rat and protected against pressure overload-induced cardiac hypertrophy in mice (21, 22). However, the effects of MA on MI/RI and the underlying mechanisms remain unclear.

In the present study, we intend to explore the potential effects of MA on MI/RI, as well as the possible signal pathway involved in hypoxia/reoxygenation (H/R) H9c2 cells, which may provide an attractive compound for use against the I/R-induced inflammation and apoptosis.

## Materials and Methods

### Cell Culture and Treatment

The rat embryonic cardiomyocyte line (H9c2) was obtained from the Cell Bank of China Science Academy and cultured in high glucose Dulbecco's modified Eagle's medium (DMEM, Gibco, MA, USA) containing 10% fetal bovine serum (FBS, HyClone, UT, USA), 100 U/ml penicillin, and 100 μg/ml streptomycin (Solarbio, China) at condition of 37°C with 5% CO_2_. The H9c2 cells were used for the experimental procedures at 60–70% confluence.

Maslinic acid was purchased from Med Chem Express (purity ≥ 98%, MCE, NJ, USA) and dissolved in dimethyl sulfoxide (DMSO). At confluence, the H9c2 cells were treated with varying concentrations of commercially available MA (5–100 μM), prepared in DMEM medium for 24 h before H/R stimulation. In the untreated control groups, the H9c2 cells were pretreated with the same amount of DMSO as that in the treated groups.

### Establishment of H/R Cell Model and Cell Grouping

The H/R model was established as previously described (23, 24). Briefly, the cells were cultured in glucose-free DMEM and then put into a near-anaerobic atmosphere (5% CO_2_ and <0.1% O_2_) produced using an Anaero Pack (Mitsubishi Gas Company, Tokyo, Japan). Followed by incubation in hypoxic conditions at 37°C for 4 h, the cells were cultured in normoxic condition (reoxygenation) for another 12 h. The cells were randomly assigned into the control group, H/R group, H/R+MA5 (H9c2 cells pretreated with 5 μM MA and subjected to H/R procedure) group, H/R+MA10 (the H9c2 cells pretreated with 10 μM MA and subjected to H/R procedure) group, and H/R+MA20 (the H9c2 cells pretreated with 20 μM MA and subjected to H/R procedure).

### Animals and Treatment

The experiments were approved by the Ethics Committee for Animal Experimental Center of Nankai University, Tianjin, China. All the experimental protocols were performed in compliance with the US National Institutes of Health guidelines. Fifty-five healthy adult Sprague Dawleys (SD) rats [weighing 220 ± 10 g, Animal License: SCXK (Hubei) 2017-0012, Center for Animal Experiment of China Three Gorges University, Hubei, China] were housed in a temperature-controlled room with 50% humidity under 12 h light/dark cycles, and free access to food and water. All the efforts were made to minimize the animal suffering and reduce the number of animals used.

Prior to myocardial ischemia/reperfusion (I/R) operation, the rats were treated with varying concentrations of MA (5, 10, and 20 mg/kg) or vehicle (the same amount of DMSO dissolved in normal saline) by intraperitoneal injection for two consecutive days. The rats were randomly assigned to the following group (=11 per group): (1) Sham group: sham-operation, (2) I/R group: myocardial ischemia/reperfusion-operation, (3) I/R + MA5: administered 5 mg/kg MA before I/R, (4) I/R + MA10: administered 10 mg/kg MA before I/R, and (5) I/R + MA20: administered 20 mg/kg MA before I/R.

### Establishment of MI/RI Rat Model

An MI/RI model was established as described previously (6). The rats were anesthetized by intraperitoneal injection of 3% sodium pentobarbital (40 mg/kg), and then ventilated with room air using a small animal ventilator. Meanwhile, the limbs of rats were connected to ECG machine (VE-300, China). Then, the hearts were exposed gradually through a left thoracotomy at the fourth intercostal space and a 6-0 silk suture over a 1-mm medical latex tube was placed around the origin of left anterior descending coronary artery (LAD). After 30 min of ischemia, the suture was cut, allowing the myocardium to be reperfused for 3 h. The Sham group was subjected to the same surgical procedure without blood occlusion of LAD. At the end of reperfusion, the rats were anesthetized and euthanized in a CO_2_ chamber, and the heart and blood specimens were collected.

### Cell Viability Assay

The viability of H9c2 cells was measured as per the instructions of Cell Counting Kit-8 (CCK-8) assay (Dojindo, Japan). The H9c2 cells were seeded in 96-well plates at a density of 5,000 cells per well. After proper treatment, the CCK-8 reagents (10 μl/well) were added into each well and incubated for additional 2 h, and then the optical density (OD) values were measured at 450 nm by a microplate spectrophotometer.

### Cell Apoptosis Analysis

The cell apoptosis was determined by Annexin V-PE/7-AAD (Becton Dickinson Co, NJ, USA) according to the instructions of the manufacturer. Approximately, 1 × 10^5^ H9c2 cells were placed on six-well plates. Followed by the two washes with PBS, the H9c2 cells were collected and analyzed using BD Accuri® C6 Plus Flow Cytometry (BD Bioscience, NJ, USA).

### Detection of Cardiac Enzymes

The levels of cardiac enzymes, such as lactate dehydrogenase (LDH) and creatine kinase isoenzyme-MB (CK-MB) in serum and culture medium, were detected by the ADVIA2400 automatic biochemical analyzer (SIEMENS, Germany).

### Enzyme-Linked Immunosorbent Assay (ELISA)

After various treatments, the levels of IL-1β, (IL-6) and TNF-α in the serum and cell culture supernate were detected by specific enzyme-linked immunosorbent assay ELISA kits (R&D Systems, MN, USA) based on the instructions of the manufacturer. The value of absorbance was detected using a microplate spectrophotometer at 450 nm. Then, the concentrations of cytokines were calculated by reference to the standard curves.

### Immunofluorescence Staining

The H9c2 cells were fixed with 4% paraformaldehyde for 15 min, blocked with 0.3% Triton X-100, incubated with the anti-NF-κB p65 antibody (CST, 1:500) at 4°C overnight, and subsequently incubated with a Cy3 secondary antibodies for 2 h at room temperature in the dark. After incubation, the cells were washed three times in PBS, and stained with 4′,6-diamidino-2-phenylindole (DAPI, Beyotime, China) for nucleus identification. The images were captured using a fluorescence microscope (Leica DMi8, Germany). The data illustrated were representative of at least three independent repeats. The relative fluorescence intensity in the nucleus (NF-κB) was analyzed by ImageJ software.

### H&E Staining

The myocardial tissue was collected and fixed with 4% paraformaldehyde and embedded in paraffin. Subsequently, all the tissue samples were cut into 6-μm sections and stained with H&E as previously described (25). Then, the pathological changes of myocardial tissue were inspected under a light microscope.

### Terminal Deoxynucleotidyl Transferase-Mediated dUTP Nick-End Labeling (TUNEL) Staining

The apoptosis of cardiomyocytes in tissue sections was assessed by terminal deoxynucleotidyl transferase-mediated dUTP nick-end labeling (TUNEL) staining (Beyotime, China) according to the instructions of the manufacturer. Briefly, a double staining technique was used: TUNEL-positive cells displayed green fluorescence and the nucleus of all cells were stained with DAPI which produced blue fluorescence. The apoptotic index was determined by the equation: TUNEL-positive cells/ DAPI positive cells × 100%.

### Measurement of Myocardial Infarct Size

The myocardial infarct size was evaluated by 2,3,5-triphenyltetrazolium chloride (TTC, Sigma-Aldrich, MO, USA) staining as previously described (6). The hearts of rats were harvested immediately after 3 h reperfusion, rinsed with saline, and frozen at −20°C for 20 min. Then, the hearts were crosscut into 1.5 mm thick slices and incubated in 1.5% TTC solution at 37°C in the dark for 15 min. Afterward, the slices were taken out, fixed with 4% paraformaldehyde overnight, and photographed. The proportion of the infarcted area (white) was obtained *via* Image-Pro Plus 7.0 software (Media Cybernetics, Inc., MD, USA).

### Western Blot Analysis

Total proteins from the H9c2 cells or rat cardiac tissues were extracted using radioimmunoprecipitation (RIPA) buffer (Beyotime, China). The protein concentration was assessed by Bicinchoninic Protein Assay Kit (Beyotime, China). All extracted proteins were boiled at 95°C in loading buffer for 5 min. Identical quantities of protein were divided by 10% sodium dodecyl-sulfate polyacrylamide gel electrophoresis (SDS-PAGE) with the voltage from 80–120 V, and then, transferred to PVDF membranes (200 mA). Afterward, the membranes were hindered with 5% skimmed milk for 2 h and hatched overnight at 4°C with the diluted primary antibodies for Bax (1:1000, Proteintech Group, China), Bcl-2 (1:1000, Proteintech Group, China), caspase-3 (1:1000, CST, MA, USA), HMGB1 (1:1000, CST, MA, USA), IκBα (1:1000, CST, MA, USA), P-IκBα (1:1000, CST, MA, USA), p65 (1:1000, CST, MA, USA), P-p65 (1:1000, CST, MA, USA), MyD88 (1:1000, CST, MA, USA), TLR4 (1:1000, Proteintech Group, China), GAPDH (1:5000, Proteintech Group, China), and β-actin (1:2000 Proteintech Group, China). After three tris-buffered saline tween (TBST) washes, the horseradish peroxidase-conjugated secondary antibodies (CST, MA, USA) were used for hatching the membranes for 1.5 h. Subsequently, an enhanced chemiluminescence detection system was used for the analysis of protein bands and gray intensity analysis was quantified using Image J software.

### Molecular Docking Simulation

The binding sites of MA into HMGB1 protein molecular was achieved using AutoDockTools-1.5.6 and Python-2.4 software. Information regarding MA and HMGB1 protein was downloaded from the Traditional Chinese Medicine Systems Pharmacology (TCMSP) database (https://old.tcmspe.com/tcmsp.php) and the Research Collaboratory for Structural Bioinformatics (RCSB) Protein Data Bank (https://www.pdbus.org/) respectively, which were saved in mol2 or pdb format. Prior to docking, the ligand and acceptor structures were preprocessed through the deleting water molecules, adding hydrogens, and Gasteiger charge to create. Then, AutoGrid program was used to construct the grid maps and AutoDock needs pre-calculated grid maps for performing the docking calculations fast. The final pose was determined based on the lowest binding energy. The results were visualized by PyMOL 3.8 software, and the hydrogen bonds as well as their binding sites were observed and analyzed.

### Recombinant HMGB1 Pre-intervention

To assess for the effect of HMGB1 on NF-κB activation by MA, a verification experiment using rHMGB1 (R&D Systems, MN, USA) was conducted. After pretreated with 20 μM MA, the H9c2 cells were treated with recombinant HMGB1 (rHMGB 1) protein (200 ng/ml) or vehicle for 2 h and then, subjected to H/R procedure. The application concentration of rHMGB1 was determined according to the previously published articles (26, 27). After various treatments, the supernatants and cells were collected to detect the concentration of inflammatory factors by ELISA and the level of apoptotic-related proteins by western blot.

### Statistical Analysis

All data were expressed as the mean ± SD, and analyzed using SPSS 20.0 software (IBM Corp., NY, USA). One–way ANOVA followed by the least significant difference (LSD) *post-hoc* test was used to analyze the differences between the groups. The value of *p* < 0.05 was considered statistically significant. The histograms were graphed using GraphPad Prism 7.00 software (San Diego, CA, USA).

## Results

### Effects of MA on H/R-Induced Cardiomyocyte Injury

**Figure 1:**
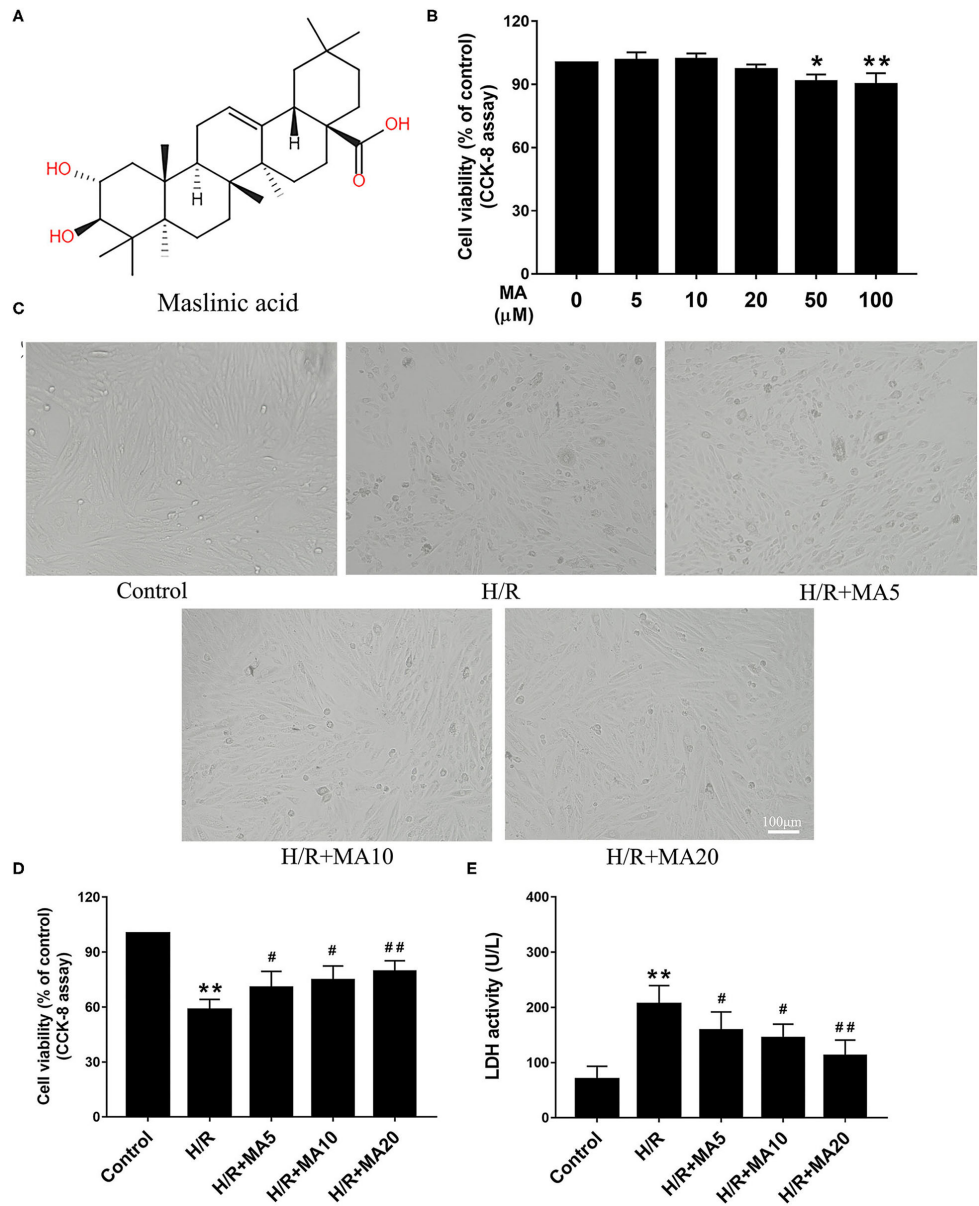
The effects of maslinic acid (MA) on hypoxia/reoxygenation (H/R)-induced cardiomyocyte injury. **(A)** Chemical structure of MA. **(B)** The cytotoxicity effects of MA on the H9c2 cells were tested by the Cell Counting Kit-8 (CCK-8). **(C)** After pretreating with indicated concentrations of MA (5, 10, and 20 μM) and being subjected to H/R procedure, the morphology of H9c2 cells were detected by a light microscope. **(D,E)** The H9c2 cells injury were measured d by CCK-8 assay and lactate dehydrogenase (LDH) release assay. All the values are representative of at least three independent experiments and expressed as the mean ± SD. *P < 0.05 and **P < 0.01 vs. control group; ^#^*P* < 0.05 and ^##^*P* < 0.01 vs. H/R group.

The chemical structure of MA is shown in Figure 1A. The cytotoxicity effects of MA on the H9c2 cells were detected at a wide range of concentrations (0, 5, 10, 20, 50, and 100 μM) for 24 h using the CCK-8 assay. As shown in Figure 1B, MA treatment led to a significant decrease in cell viability when the concentration of MA reached 50 μM (*P* < 0.05) and 100 μM (*P* < 0.01). Thus, 5, 10, and 20 μM MA were utilized for the following experiments. It is well known that H/R, a reliable model for *in vitro* to mimic I/R, is widely used in the MI/RI research and the development of cardiovascular drugs. The H/R model of the cardiomyocytes can be directly observed the morphological structure, physiological, and biochemical indexes of the injured cardiomyocytes (24, 28). Then, the H9c2 cells were pre-incubated with MA for 24 h and subjected to H/R.

The cell morphology was detected by a light microscope. After H/R injury, the H9c2 cells lost their elongated spindle-shape morphology, exhibiting round, shrunken, and typical apoptotic morphologies, such as blebbing, apoptotic bodies, and detachment, while 5, 10, and 20 μM of MA treatment could attenuate the above changes in the cell morphologies (Figure 1C). Based on the above results, we subsequently proposed to explore the effects of MA on the cell viability and LDH release after H/R injury. The CCK-8 assay results showed that the cell viability was significantly decreased in the H/R group, compared with the control group (Figure 1D). Conversely, MA treatment increased the cell viability after H/R injury (5, 10, and 20 μM of MA vs. H/R: 70.5 ± 5.8%, 74.7 ± 8.9%, 79.4 ± 5.9% vs. 58.2 ± 5.8%), showing a dose-dependent relationship (Figure 1D). Additionally, these results were confirmed by LDH release assay. As shown in Figure 1E, the level of LDH in the H/R group was markedly increased relative to the control group, whereas MA treatment (5, 10, and 20 μM) in a dose-dependent manner strongly reduced the H/R-induced LDH release. Thus, these results indicated that MA attenuated H/R-induced cardiomyocyte injury.

### The Effects of MA on Cardiomyocyte Apoptosis and Expression of Apoptosis-Related Proteins After H/R Treatment

**Figure 2:**
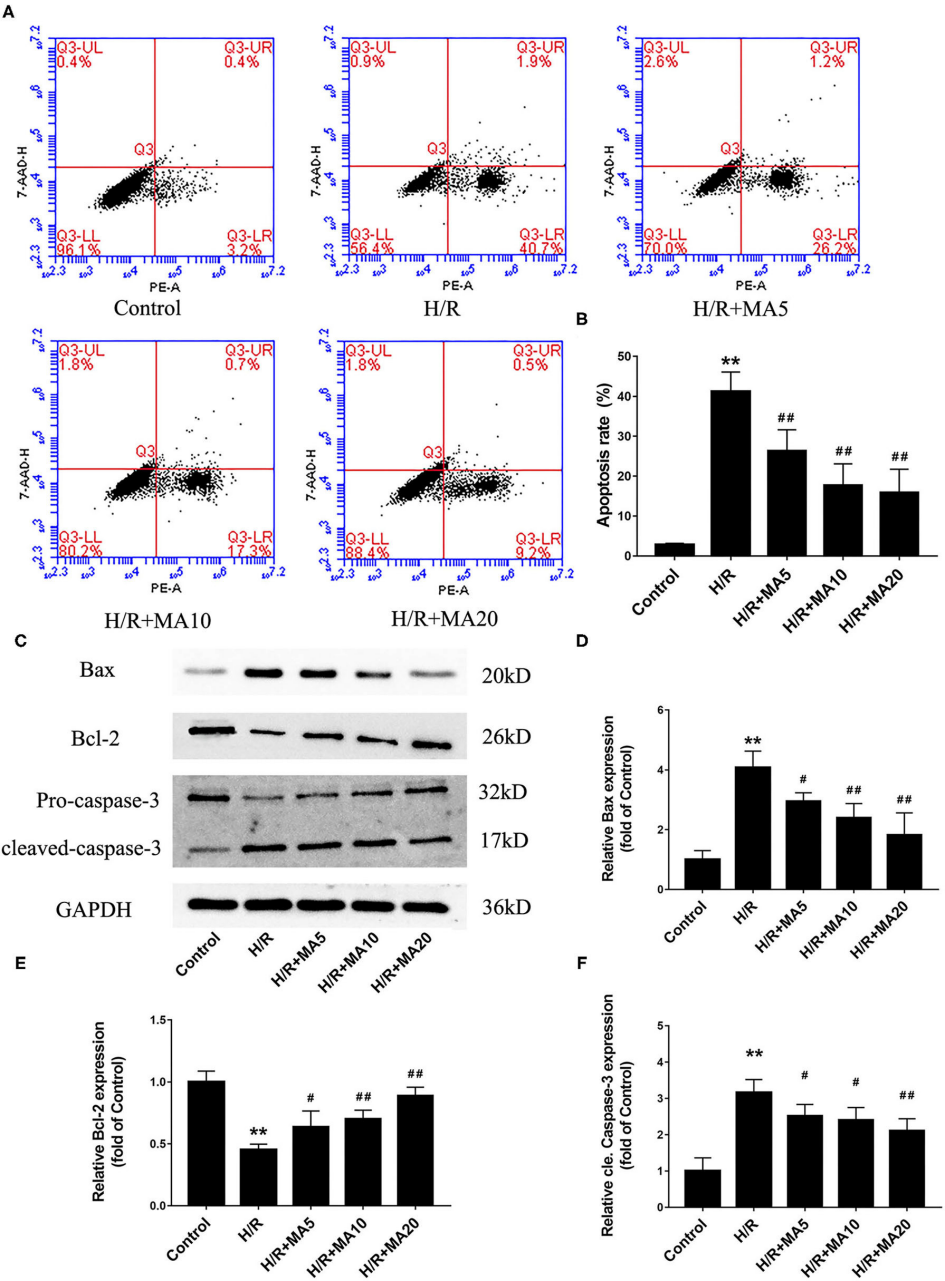
The effects of MA on cardiomyocyte apoptosis and the expression of apoptosis-related proteins after H/R treatment. **(A,B)** After various treatments, the apoptosis is measured by Annexin V-PE/7-AAD using flow cytometry and the results are expressed in bar graphs. **(C)** Western blot was performed to assess the expression of apoptosis-related proteins (Bax, Bcl-2, and cleaved caspase-3) in H9c2 cell under H/R conditions. **(D–F)** The quantitative analysis of apoptosis-related proteins is shown in bar graphs. All the values are representative of at least three independent experiments and expressed as the mean ± SD. **P < 0.01 vs. control group; ^#^*P* < 0.05 and ^##^*P* < 0.01 vs. H/R group.

In our previous study, we confirmed that apoptosis contributes to cardiomyocyte death during the MI/RI (6). So there and then, we measured the effects of MA on H/R-induced apoptosis by flow cytometric analysis. As shown in Figures 2A,B, the percentage of apoptotic H9c2 cells was obviously increased in H/R group, compared with the control group (2.8 ± 0.4% vs. 41.2 ± 4.9%). Instead, 5, 10, and 20 μM MA treatment significantly reduced the apoptosis rate to 26.3 ± 5.3, 17.7 ± 5.4, and 15.9 ± 5.8%, respectively. Next, we further examined the expressions of several apoptosis-related proteins by western blotting analysis (Figures 2C–F). Compared with the control group, the cleaved caspase-3 and Bax protein expression were significantly increased, while the Bcl-2 protein expression was obviously decreased in the H/R group. Conversely, MA treatment (5, 10, and 20 μM) obviously reduced the levels of cleaved caspase-3 and Bax expression, while increased Bcl-2 expression in a dose-dependent manner after H/R.

### The Effects of MA on Inflammatory Cytokines in Cell Supernatants and the NF-κB Nuclear Translocation After H/R Treatment

**Figure 3:**
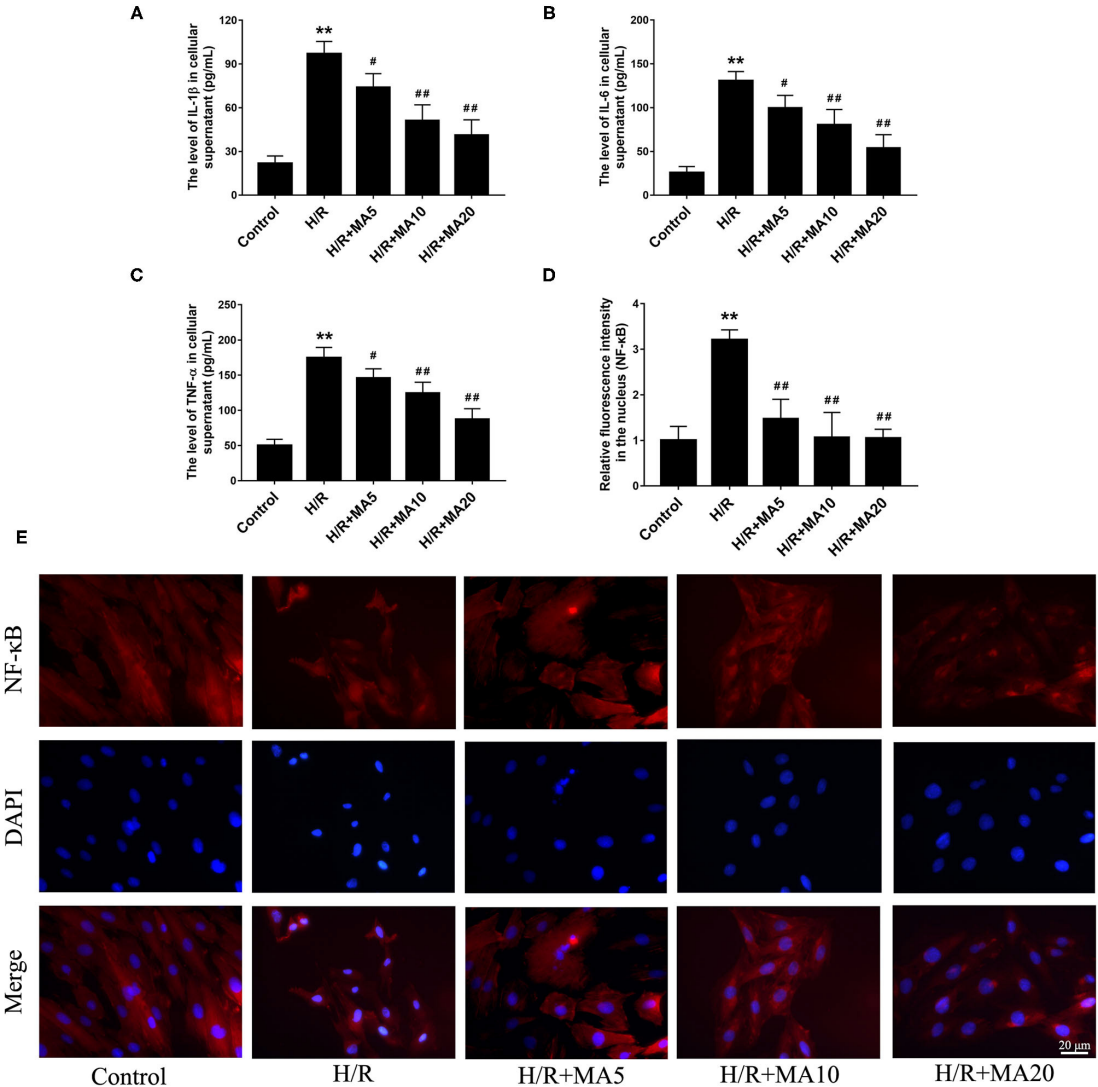
The effects of MA on the inflammatory cytokines in cell supernatants and the NF-kB nuclear translocation after H/R treatment. **(A–C)** The levels of interleukin 1 β (IL-1β), interleukin 6 (IL-6), and tumor necrosis factor alpha (TNF-α) in the cell supernatants were assessed by the specific enzyme-linked immunosorbent assay (ELISA) analyses. **(D,E)** The nuclear translocation of nuclear factor-κB (NF-κB) p65 in a H9c2 cell was investigated by immunofluorescence staining. NF-κB p65 (red), 4′,6-diamidino-2-phenylindole (DAPI)-labeled nuclei of cardiomyocytes (blue) and merged images show NF-κB translocation into the nucleus (purple). The quantification analysis of the mean fluorescent intensity in the nucleus is shown in bar graphs. All the values are representative of at least three independent experiments and expressed as the mean ± SD. **P < 0.01 vs. control group; ^#^*P* < 0.05 and ^##^*P* < 0.01 vs. H/R group.

To determine whether MA could inhibit the inflammatory cytokines, we analyzed the expression of IL-1β, IL-6, and TNF-α in cell supernatants during H/R. The results from the ELISA analyses (Figures 3A–C) showed that the levels of IL-1β, IL-6, and TNF-α were significantly increased in the H/R group, relative to the control group. While compared with the H/R group. MA treatment (5, 10, and 20 μM) markedly reduced the levels of IL-1β, IL-6, and TNF-α in a dose-dependent manner.

An NF-κB, a nuclear transcription factor, acts as an important mediator of apoptosis and inflammatory response (29). We performed the immunofluorescence staining to observe the nuclear translocation of NF-κB p65. As shown in Figures 3D,E, H/R treatment obviously promoted the translocation of p65 into nucleus compared with the control group, and administration of MA (5, 10, and 20 μM) significantly blocked the nuclear translocation of p65 after H/R. All the above results suggested that MA could inhibit the H/R-induced cardiomyocyte apoptosis and inflammation, which was related to NF-κB transcription.

### Effect of MA on HMGB1-TLR4-MyD88-NF-κB Pathway After H/R Treatment

**Figure 4:**
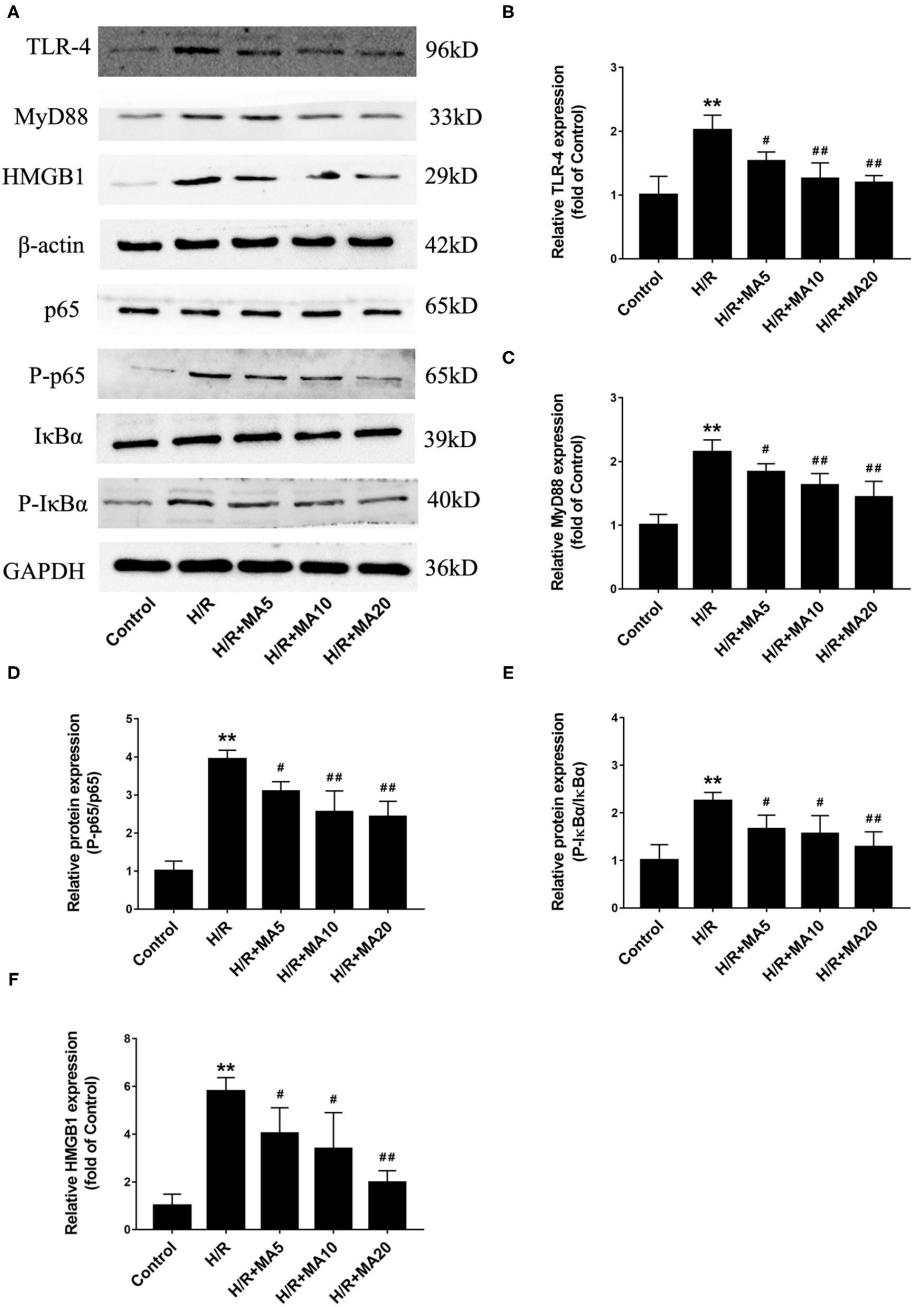
Effect of MA on high mobility group box 1 (HMGB1)- toll-like receptor 4 (TLR4)- myeloid differentiation factor 88 (MyD88)-NF-κB pathway after H/R treatment. **(A)** Western blotting was performed to assess the expression of HMGB1, TLR4, MyD88, p65, P-p65, IκBα, and P-IκBα in H9c2 cell under H/R conditions. **(B–F)** The quantitative analyses of HMGB1, TLR4, MyD88, P-p65/p65, and P-IκBα/IκBα are shown in bar graphs. All the values are representative of at least three independent experiments and expressed as the mean ± SD. **P < 0.01 vs. control group; ^#^*P* < 0.05 and ^##^*P* < 0.01 vs. H/R group.

To determine the mechanism by which MA suppressed the inflammation and apoptosis in MI/RI, we analyzed the effects of MA on the signaling pathways involving HMGB1, a vital mediator in the pathophysiology of the MI/RI through TLR4-MyD88-NF-κB mediated signaling, and may represent an important therapeutic target (12, 15). As shown in Figures 4A–F, the protein levels of HMGB1, TLR4, MyD88, and the phosphorylation of NF-κB p65 and IκBα were markedly upregulated in the H/R group compared with those in the control group. However, 5, 10, and 20 μM of MA treatment markedly suppressed the protein expression of HMGB1, TLR4, MyD88, and the phosphorylation of NF-κB p65 and IκBα in a dose-dependent manner after H/R. Therefore, our results suggested that MA could inhibit the H/R-induced activation of HMGB1-TLR4-MyD88-NF-κB signaling pathway.

### MA Attenuated H/R-Induced Inflammation and Apoptosis *via* Regulating HMGB1

**Figure 5:**
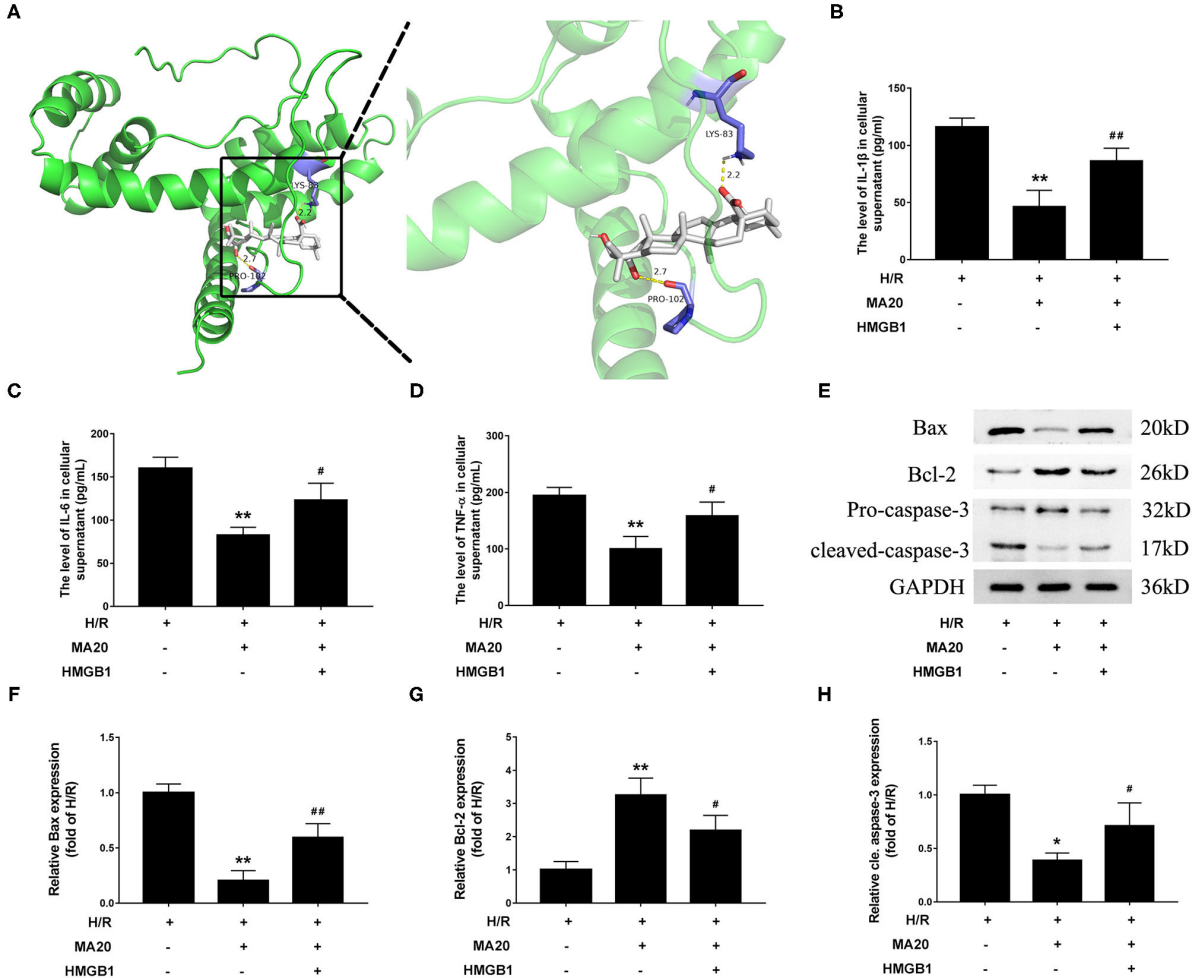
Maslinic acid attenuated H/R-induced inflammation and apoptosis *via* regulating HMGB1. **(A)** Final three-dimensional homology structure predictions of the docking conformation between MA and HMGB1. The sticks are shown for catalytic residues LYS-83 and PRO-102 (purple). The hydrogen bond (dashed yellow lines) distances are 2.2 and 2.7. **(B–D)** After pretreated with 20 μM of MA or plus rHMGB1, the levels of IL-1β, IL-6 and TNF-α in culture medium were assessed by the specific ELISA analyses under H/R conditions. **(E)** Western blot was performed to assess the expression of apoptosis-related proteins (Bax, Bcl-2, and cleaved caspase-3) in H9c2 cell under H/R conditions. **(F–H)** The quantitative analysis of the apoptosis-related proteins is shown in bar graphs. All the values are representative of at least three independent experiments and expressed as the mean ± SD. *P < 0.05 and **P < 0.01 vs. H/R group; ^#^*P* < 0.05 and ^##^*P* < 0.01 vs. H/R + MA20 group.

Through the molecular docking experiments, it was found that MA and HMGB1 were docked in the predicted binding sites. The docking simulations yielded ideal binding conformation with the lowest binding free energy of −8.95 kcal/mol and formed two hydrogen bonds (Figure 5A). For further confirming that MA attenuated H/R-induced inflammation and apoptosis *via* regulating HMGB1, a verification experiment using rHMGB1 was conducted. As shown in Figures 5B–D, 20 μM of MA treatment significantly inhibited the release of proinflammatory factors during H/R, such as IL-1β, IL-6, and TNF-α, related to the H/R group. However, co-incubation with rHMGB1 abolished the inhibitory effects of MA on the release of IL-6, IL-1β, and TNF-α. As the same time, compared with the H/R group, 20 μM of MA treatment also markedly suppressed the level of cleaved caspase-3 and Bax expression, while these aforementioned effects of MA were reversed by the rHMGB1 treatment (Figures 5E–H). These results indicated that the cardioprotective effect of MA on inflammation and apoptosis after H/R might, at least partly, be due to the inhibition of HMGB1 axis.

### MA Treatment Ameliorated I/R-Induced Myocardial Injury and Inflammation *in vivo*

**Figure 6:**
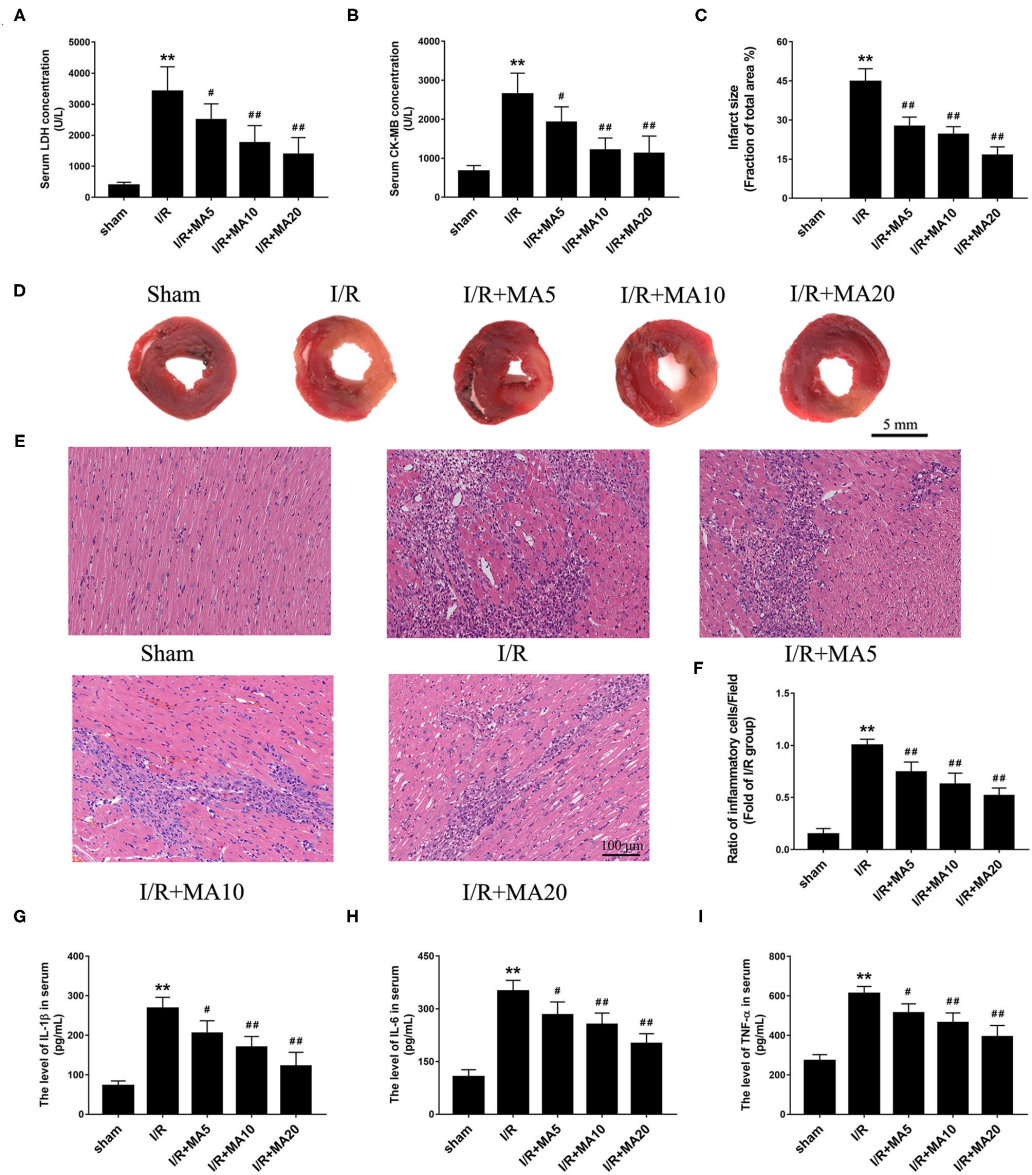
Maslinic acid treatment ameliorated I/R-induced myocardial injury and inflammation *in vivo*. **(A,B)** After pretreating with indicated concentrations of MA (5, 10, and 20 mg/kg) and being subjected to I/R procedure, the myocardial injury was detected by LDH and CK-MB release assay (*n* = 4). **(C,D)** Myocardial infarction was detected by 2,3,5-triphenyltetrazolium chloride (TTC) staining and the infarct size was analyzed as (white area)/(white area + red area). The representative photographs of TTC staining performed in the graph (*n* = 4). **(E,F)** Myocardial histology and inflammatory cell infiltration are assessed by H&E staining and the results of inflammatory cell infiltration (%) within the ischemic heart are expressed in the bar graphs (*n* = 4). **(G–I)** The levels of IL-1β, IL-6, and TNF-α in serum was assessed by the specific ELISA analyses (*n* = 3). All data are expressed as mean ± SD. **P < 0.05 vs. control group; ^#^*P* < 0.05, and ^##^*P* < 0.01 vs. H/R group.

An MI/RI rat model was established to further confirm the effect of MA *in vivo*. As shown in Figures 6A,B, the level of LDH and CK-MB in serum was dramatically increased after MI/RI, compared with the sham group. As expected, MA treatment (5, 10, and 20 mg/kg) obviously decreased I/R injury-aroused the release of LDH and CK-MB in a dose-dependent manner. Then, we examined the myocardial infarct size using TTC staining (Figures 6C,D). The results showed that the infarct size was significantly increased to 44.7 ± 5.0% in I/R group, whereas MA treatment (5, 10, and 20 mg/kg) significantly reduced I/R injury-caused infarct size to 27.5 ± 3.6, 24.5 ± 3.0, and 16.4 ± 3.3%, respectively, showing a dose-dependent relationship.

To further examine the role of MA on I/R-induce inflammation *in vivo*, H&E staining and ELISA analysis was performed. As shown in Figures 6E,F, compared with Sham group, I/R obviously increased the infiltration of inflammatory cells into the injured myocardium. However, 5, 10, and 20 mg/kg of MA treatment significantly decreased the infiltration of inflammatory cells in I/R myocardium. Meanwhile, we also measured the concentrations of pro-inflammatory cytokine IL-1β, IL-6 and TNF-α in serum (Figures 6G–I). The results showed that the levels of IL-1β, IL-6 and TNF-α were dramatically elevated in serum after MI/RI relative to the sham group. As expected, MA treatment (5, 10, and 20 mg/kg) obviously reduced the release of IL-1β, IL-6, and TNF-α in serum after MI/RI, which was consistent with the results *in vitro*. These data suggested that MA preconditioning attenuated I/R-induced myocardial injury and inflammation.

### MA Treatment Suppressed I/R-Induced Myocardial Apoptosis *in vivo*

**Figure 7:**
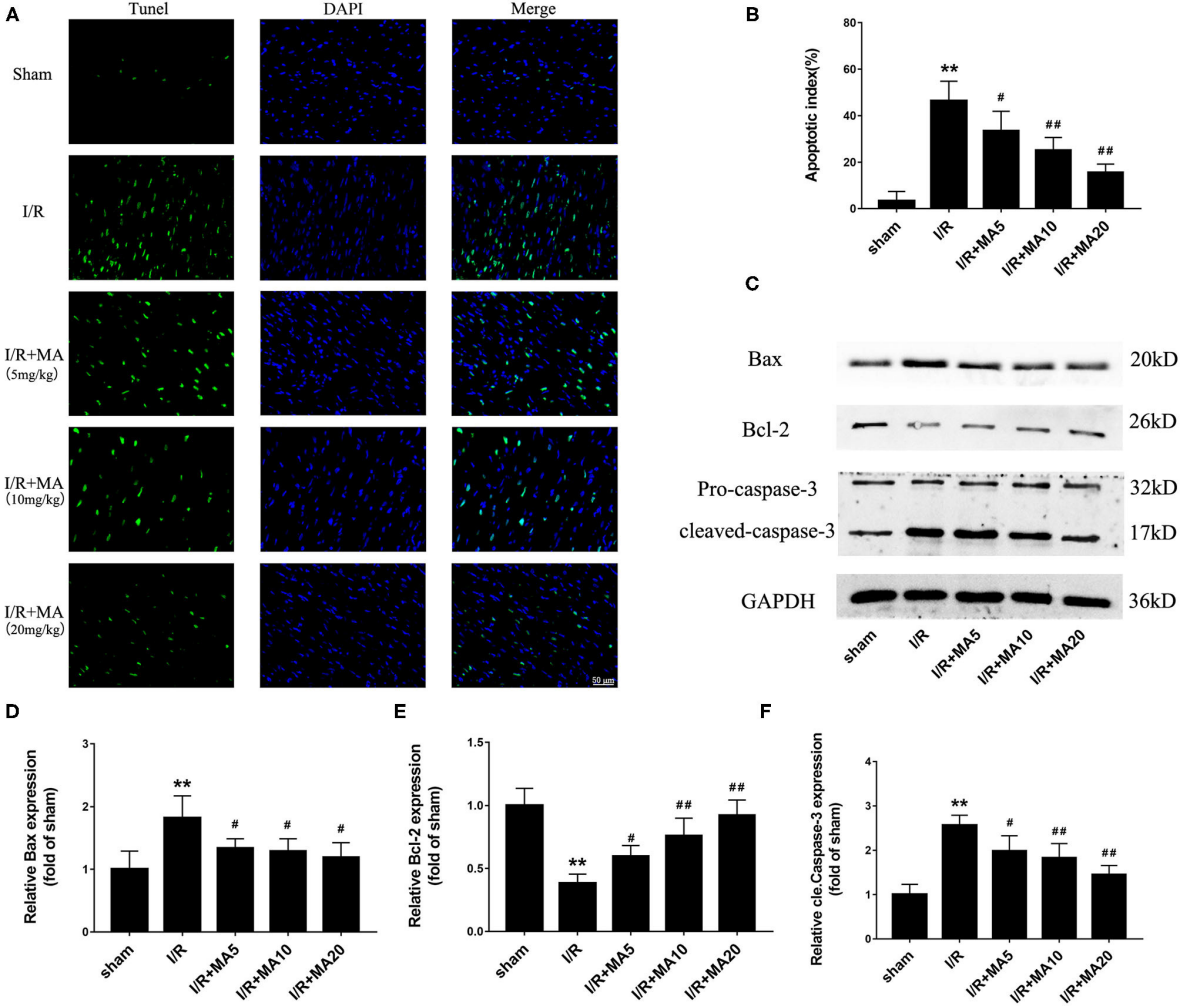
Maslinic acid treatment suppressed I/R-induced myocardial apoptosis *in vivo*. **(A,B)** Terminal deoxynucleotidyl transferase-mediated dUTP nick-end labeling (TUNEL) staining was used to assess the myocardial apoptosis and green fluorescence of the nucleus is indicative of apoptosis. The quantitative analysis of the mean apoptotic index is shown in the bar graphs (*n* = 4). **(C)** Western blot was performed to assess the expression of apoptosis-related proteins (Bax, Bcl-2, and cleaved caspase-3) in myocardial tissue under I/R conditions. **(D–F)** The quantitative analyses of the apoptosis-related proteins are shown in bar graphs (*n* = 3). All data are expressed as mean ± SD. **P < 0.05 vs. control group; ^#^*P* < 0.05, and ^##^*P* < 0.01 vs. H/R group.

Next, to further examine the role of MA on I/R-induce apoptosis *in vivo*, we used TUNEL staining and detected the expression of apoptosis-related proteins. As shown in Figures 7A,B, the apoptotic cells were increased to 46.4 ± 8.5% in the I/R group compared with the sham group, while 5, 10, and 20 mg/kg of MA treatment significantly reduced the number of apoptotic cells to 33.5 ± 8.4%, 25.1 ± 5.5%, and 15.6 ± 3.6% in I/R myocardium, showing a dose-dependent relationship. Moreover, the results of the western blot (Figures 7C–F) indicated that I/R markedly increased the expression of Bax, and cleaved caspase-3 while decreased the Bcl-2 expression. As expected, MA treatment (5, 10, and 20 mg/kg) obviously reduced the levels of Bax and cleaved caspase-3 expression, while elevated the level of Bcl-2 expression (Figures 7C–F). Above results showed a similar trend to that obtained in the H/R H9c2 cells. Thus, these results indicated that MA preconditioning attenuated I/R-induced myocardial injury by inhibiting myocyte apoptosis *in vivo*.

## Discussion

Over the past two decades, accumulating evidence indicates that MI/RI is an extremely complex pathological event with many interlinked processes involving inflammatory response, cardiomyocyte apoptosis, oxidative stress, and calcium overload (6, 9, 28, 30). Nevertheless, to date, there has been no effective strategy for preventing or limiting MI/RI. In this study, we presented first evidence that MA, as a novel cardioprotective agent, against I/R-induced myocardial injury by inhibiting the inflammatory response and cardiomyocyte apoptosis both *in vivo* and *in vitro*. Meanwhile, we observed that the cardioprotective effect of MA on the myocardial injury was mainly dependent on HMGB1/TLR4/MyD88/NF-κB signaling pathway. Thus, these findings not only demonstrate the important role of MA in MI/RI, but also reveal the novel mechanism of MA attenuating I/R-induced inflammation and apoptosis.

In recent years, MA, as an NF-κB inhibitor, has been extensively studied for its anti-inflammatory, antioxidant, and anti-tumor activities (17, 18, 22). Lee et al. (18) reported that MA suppressed the inflammatory response *via* the inhibition of NF-κB and STAT-1 in an LPS-induced lung injury. Correspondingly, Chen et al. (31) showed that MA attenuated IL-1β-induced inflammatory response in osteoarthritis by inactivating the PI3K/AKT/NF-κB pathway, which might be used as a safe and effective potential therapeutic strategy for osteoarthritis. Meanwhile, through the dorsal skinfold chamber model to analyze the effects of MA on I/R-induced inflammation, study by Ampofo et al. (32) found that MA alleviated I/R-induced tissue injury and inflammation *via* suppressing the NF-κB-mediated adhesion molecule expression. Interestingly, MA has demonstrated certain advantages in the treatment of cardiovascular disease. MA could protect the myocardium from injury caused by the diabetic cardiomyopathy *via* anti-glycative and anti-coagulatory activities in mice (33). Then, during pressure overload-induced cardiac hypertrophy, MA treatment decreased the hypertrophic markers, improved the cardiac function, and restricted cardiac fibrosis by inhibiting the activation of protein kinase B (AKT) and extracellular regulated protein kinases (ERK) signaling pathway (21). Additionally, Shaik et al. (22) revealed that MA offered cardioprotection on ISO-induced MI through the inhibition of lipid peroxidation and oxidative stress. Taken together, these results indicate that MA has exerted potent anti-inflammatory effects along with antioxidant properties in multiple types of cells, showing preclinical promise across a wide range of diseases. The myocardial injury-related enzyme LDH can effectively reflect the degree of myocardial damage. Under the physiological conditions, the extracellular and serum LDH levels are extremely low, and LDH can be released from the damaged cells into the culture medium and serum after MI/RI. In the current study, our data showed that MA treatment in a dose-dependent manner reduced the area of MI and the levels of the LDH after I/R injury by suppressing the inflammation and apoptosis. Notably, the single oral administration of the MA at 1,000 mg/kg or the repeated daily oral administration of 50 mg/kg of MA for 28 days to mice did not produce any signs of morbidity and toxicity during the experimental period (34). Similarly, the lack of harmful effects found in the hematology, clinical biochemistry, and histopathology evaluation indicated that MA administered group has a large safety margin (34). Thus, given its broadly distributed and multiple bioactivities in natural sources, a suitable food or drug-containing MA treatment is worthy of being recommended for use in clinical applications.

Apoptosis, a major type of programmed cell death, is one of the main features in the pathophysiology of MI/RI, leading to cardiomyocyte loss, myocardium remolding, and the aggravation of the inflammatory response (35, 36). Then, Bax and Bcl-2 are the important mediator and regulator of cell apoptosis, and ultimately activates caspase-3, which is the apoptosis executor, causing proteolysis. On another hand, I/R occurring in the cardiomyocytes induces an inflammatory cascade, leading to further damage to the myocardium. The pathogenesis involves the infiltration of inflammatory cells and the production of inflammatory cytokines, such as TNF-α, IL-1β and IL-6. It is well known that NF-κB transcription factor played a critical role in the regulation of inflammation and cell death in cardiac pathology (37, 38). NF-κB activation depended on IκB kinase (IKK)-mediated phosphorylation IκB-α which is then released from the NF-κB complex, and subsequently activated NF-κB p65 translocation to the nucleus, where phosphorylated p65 regulated the expression of target gene (29). In the present study, MA decreased the release of inflammatory cytokines and the expression of apoptosis-related protein after MI/RI both *in vitro* and *in vivo*. Consistently, MA could restrain degradation of IκB-α, phosphorylation of p65, and nuclear translocation of p65, which contributed to the inactivation of NF-κB signaling pathway. Briefly, the current study provides a preliminary evidence that MA treatment alleviates MI/RI *via* the regulating activity of NF-κB pathway to inhibit the inflammatory response and apoptosis.

Notably, several studies have demonstrated that MA possessed antioxidative activity on variety of tissues or cells, such as macrophage, the vascular smooth muscle cells (VSMCs), and the breast cancer cells (17, 32, 39, 40). MA affected reactive oxygen species (ROS) generation, resulting in an attenuated oxidative DNA damage. Qin et al. (39) indicated that MA protected VSMCs from the oxidative stress by enhancing the Heme oxygenase-1 (HO-1) activity. Interestingly, it has been proposed that oxidative stress after MI/RI causes aberrant ROS accumulation, aggravating the myocardial damage (28). Our latest findings suggested that increasing HO-1 enzyme activities suppressed the oxidative stress both *in vivo* and *in vitro*, and ultimately improved MI/RI (41). Besides, our unpublished data show that MA could also reduce I/R-induced oxidative stress through suppressing ROS over accumulation. Therefore, there is possibility that MA treatment may ameliorate MI/RI by regulating the oxidative stress response. The detailed pathways and mechanism, however, need to be further explored in the future.

As the primary receptors for pattern recognition, TLRs, especially TLR4, is a common upstream sensor and acts as the key mediator in the multiple intracellular pathways (14, 24). The previous findings have confirmed that TLR4 plays an important regulatory role in MI/RI (14). As a canonical downstream adaptor, MyD88 is closely involved in the inflammatory and apoptosis signaling pathways mediated by the TLR4 (5, 14, 42). Our previous studies, as well as those of others, have shown that blocking the TLR4/MyD88 signaling pathway could reduce the inflammatory response and cardiomyocyte apoptosis by inhibiting NF-κB activation and translocation into the nucleus during MI/RI (5, 15, 42). In addition, HMGB1, a highly conserved DNA binding protein, also plays a variety of roles in heart disease according to its cellular localization (43). In the nucleus, it contributes to nuclear transcription, recombination, DNA replication, and repair, while in the cytoplasm or extracellular phase, it leads to the extensive inflammatory response and multiple organ dysfunction (12). The studies have found that myocardial HMGB1expression is upregulated soon after ischemia and remains high several days after reperfusion (12, 43). Meanwhile, HMGB1 is also a specific ligand of TLR4, which could promote the inflammatory response and apoptosis during MI/RI through interacting with TLR4 and leading to NF-κB activation (11, 13). An emerging evidence indicated that MA reduced the release of HMGB1 in the LPS-activated human umbilical vein endothelial cells and inhibited the HMGB1-stimulated activation of NF-κB as well as the production of IL-1β, IL-6, and TNF-α (20). The results of this study showed that MA successfully decreased the expression of the HMGB1/TLR4 axis which might in turn, lead to the suppression of NF-κB activation. Strikingly, the molecular docking analysis showed that there were binding sites between MA and HMGB1, implicating an interaction of them. At the same time, the cardioprotective effects of MA on the inflammation and apoptosis after MI/RI were considerably restrained by rHMGB1 preconditioning. Taken together, it was initially proved that MA treatment may attenuate I/R-induced myocardial injury by modulating of NF-κB activity, at least in part *via* suppressing the HMGB1/TLR4 axis.

However, there are several limitations of this study. First, we used the H9c2 cells instead of primary cardiomyocytes for *in vitro* study. Although the H9c2 cell line has been generally accepted as a suitable model for cardiomyocytes, it lacks the morphological properties and does not accurately represent the cardiomyocytes. Second, our study showed that the cardioprotective effects of MA were dose-dependent in MI/RI, and yet it has not been established as to whether a time-dependent relationship between myocardial injury and MA concentration. Finally, as mentioned above, MA possessed anti-oxidative properties and reduced ROS accumulation. Thus, the antioxidant effect of MA involved in other molecules or pathways during MI/RI deserves further study.

In summary, our study provides the evidence that MA treatment could alleviate MI/RI through suppressing both the inflammation and apoptosis in a dose-dependent manner, and the cardioprotective effect of MA may be partly attributable to the inactivation of HMGB1/TLR4/NF-κB pathway. The manipulation of MA-mediated HMGB1 stability may offer a new therapeutic strategy for MI/RI.

## Data Availability Statement

The raw data supporting the conclusions of this article will be made available by the authors, without undue reservation.

## Ethics Statement

The animal study was reviewed and approved by the Ethics Committee for Animal Experimental Center of Nankai University.

## Author Contributions

QL and CL conceived and designed the experiments. QL, ZL, QC, and YiW performed the experiments. YaW, JF, and XY collected and analyzed the data. MX and TL operate software and analyzed the result. All authors contributed to manuscript preparation and revision.

## Funding

This work was supported by the National Natural Science Foundation of China (81970303).

## Conflict of Interest

The authors declare that the research was conducted in the absence of any commercial or financial relationships that could be construed as a potential conflict of interest.

## Publisher's Note

All claims expressed in this article are solely those of the authors and do not necessarily represent those of their affiliated organizations, or those of the publisher, the editors and the reviewers. Any product that may be evaluated in this article, or claim that may be made by its manufacturer, is not guaranteed or endorsed by the publisher.
